# Supplementary material for: Association between oral microbiome and seven types of cancers in East Asian population: a two-sample Mendelian randomization analysis
Source: Front Mol Biosci. 2023 Nov 21;10:1327893. doi: 10.3389/fmolb.2023.1327893 (PMC10702768; doi:10.3389/fmolb.2023.1327893)
Supplement: Supplementary file 1 [file DataSheet1.ZIP › Supplementary Materials/MR plots for tongue/tongue═╝/Gastric cancer/pheno.1776_to_gastric cancer_forest.pdf]

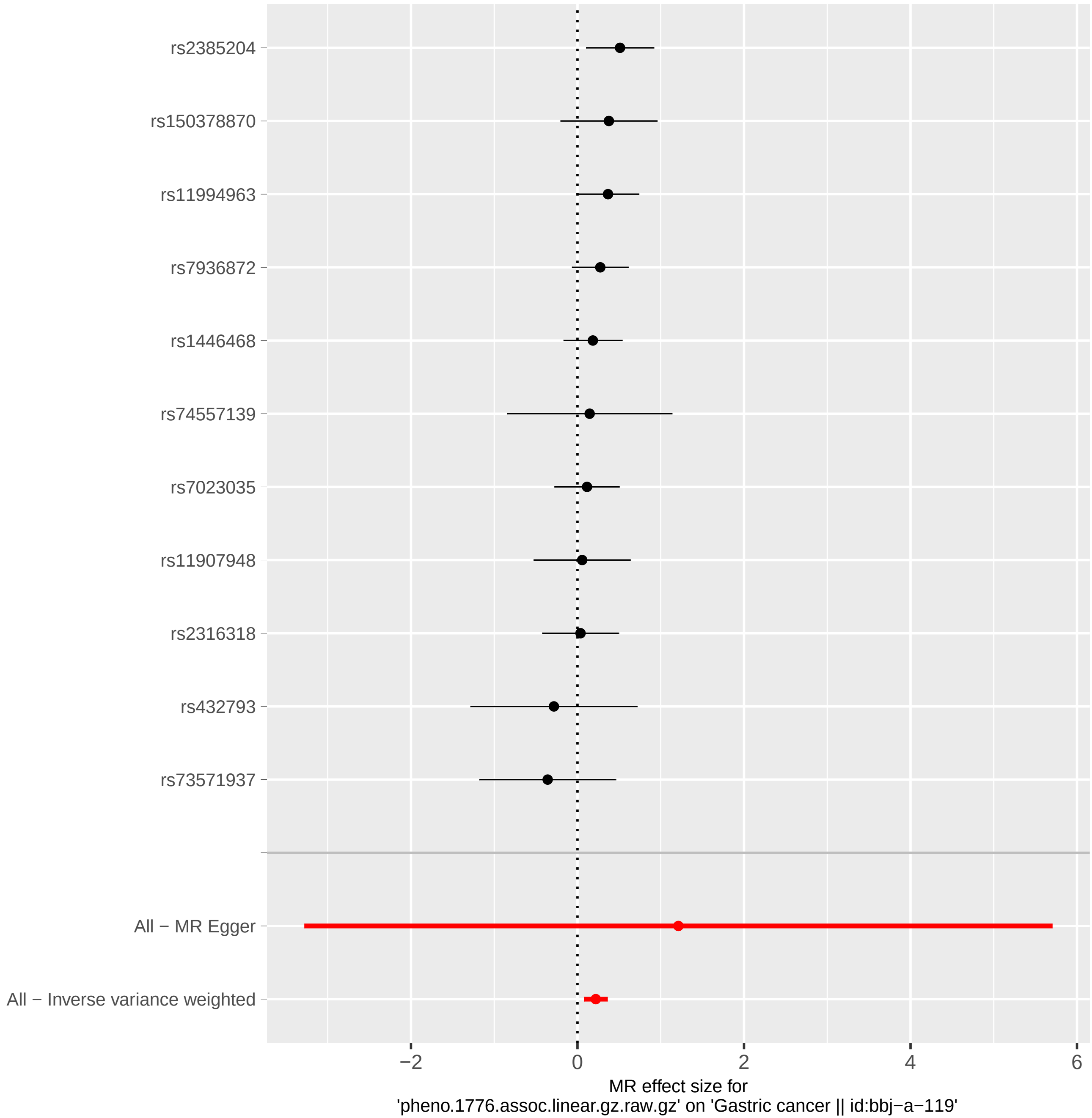

rs2385204
rs150378870
rs11994963
rs7936872
rs1446468
rs74557139
rs7023035
rs11907948
rs2316318
rs432793
rs73571937
All − MR Egger
All − Inverse variance weighted
−2
0
2
4
6
MR effect size for
'pheno.1776.assoc.linear.gz.raw.gz' on 'Gastric cancer || id:bbj−a−119'
